# Teaching inclusion health principles: What medical students must learn to be prepared to work with marginalized populations

**DOI:** 10.3389/fpubh.2025.1696499

**Published:** 2026-01-09

**Authors:** Ben King, Carlos Fuentes, Mathew Mendoza, Geraldo Medrano, David S. Buck

**Affiliations:** 1 Department of Health Systems and Population Health Sciences, Tilman J. Fertitta Family College of Medicine, University of Houston, Houston, TX, United States; 2 Humana Integrated Health Systems Sciences Institute, University of Houston, Houston, TX, United States; 3 Houston Methodist, Family Medicine Residency, Denver Harbor Clinic, Houston, TX, United States; 4 Office of Community Health, Tilman J. Fertitta Family College of Medicine, Houston, TX, United States

**Keywords:** inclusion health, medical education, primary care, cultural humility, equity, marginalized, underserved, vulnerable

## Abstract

**Introduction:**

Traditional medical education emphasizes biomedical sciences but often underprepares learners for the challenges of working with medically underserved and excluded populations. An Inclusion Health elective was developed to integrate training in the social determinants of health and the skills necessary to provide high-quality care in low-resource environments.

**Pedagogical frameworks:**

The course draws on five complementary frameworks: service learning, problem-based learning, case-based learning, cultural humility, and experiential learning, to deepen students’ understanding of exclusion in health care and to equip them with both clinical and relational skills. Together, these frameworks collectively foster critical reflection, adaptability, and a systems-level perspective.

**Learning environment:**

The elective combines classroom seminars, community-based clinical experiences, and applied learning assignments. Students work alongside outreach teams, healthcare for the homeless clinics, and social service organizations, developing practical skills in environments with limited referral networks and constrained resources.

**Assessment:**

Evaluation includes reflective writing, clinical supervision evaluations, faculty and community partner feedback, and concludes with a standardized patient assessment and reflection session, measuring cognitive and affective learning outcomes.

**Discussion:**

The program demonstrates how integrating theory-driven pedagogy with immersive, community-engaged learning can address critical educational gaps, preparing future clinicians to navigate and challenge structural inequities.

**Acknowledgment of constraints:**

Implementation faced challenges, including scheduling within the medical curriculum, sustaining community partnerships, and evaluating long-term impacts on student practice patterns.

## 1 Introduction

The traditional emphasis of undergraduate medical education on the biomedical sciences, while important, leaves many students inadequately prepared for the unique challenges faced when working with medically underserved communities (sometimes referred to as excluded populations). Consequently, there has been an ongoing, conscious shift in medical education to acknowledge the effects of the social determinants of health (SDOH) on patients and integrate these concepts within curricula that can equip learners with the skills necessary to address existing care gaps. This shift also requires a deeper understanding of the historical and ongoing exclusion of these communities from equitable healthcare access, as well as the systemic factors that sustain these inequities (1–3). Equally important to developing hard skills is cultivating soft skills—such as adaptability, cultural humility, and resourcefulness—needed to deliver high-quality care in settings that often operate with fewer resources, limited referral networks, and unique logistical constraints.

Despite the growing recognition of the importance of these qualities, a training gap exists in the teaching and learning of the skills necessary to address these communities’ multifaceted needs (4). The failure to provide this training is particularly evident among patients experiencing homelessness, whose immediate needs (food, shelter, etc.) and adverse impacts on health are not well addressed in the traditional medical model. Without addressing this training gap, well-meaning physicians may inadvertently perpetuate the health disparities that providers and healthcare systems must seek to alleviate. For example, a physician can accurately diagnose a medical illness like Alzheimer’s disease early on. However, the critical need for stabilizing housing and applying for disability income benefits to stabilize the precarious social environment of someone from a marginalized population is as important to the preservation of health as an early diagnosis.

The time pressures under which these teachings can be integrated within curricula necessitate innovative approaches to maximize adoption among our future physicians. The model of cultural humility is one framework that can be integrated through which future physicians are tasked with developing a portfolio of lifelong learning that requires reflection, as opposed to traditional learning modules and competencies. While competency-based approaches fail to account for the intersectionality and complexity in caring for excluded populations, cultural humility equips physicians with a feedback loop that values a less rigid approach to care (5, 6). This approach is critical for students who aspire to work with our most vulnerable populations.

The model of cultural humility creates a framework through which future physicians can build a portfolio of lifelong learning that requires reflection rather than being limited to external learning modules or competencies (7). Competency-based approaches often fail to incorporate and contextualize lessons for lifelong, adult learning and the related, continual need to internalize adaptive learning processes to remain up to date with the literature needed to be a good clinician. This approach is critical for students who aspire to work with our most vulnerable populations (8, 9). Unfortunately, students interested in working in these environments are often faced with a secondary, mostly informal, phase of their training to develop the necessary skill sets. Thus, they must go beyond the established curriculum to adequately prepare themselves.

Recognizing this unmet need and the importance of bridging the gap, faculty at the Tilman J. Fertitta Family College of Medicine (TJFFCOM) designed and launched “Inclusion Health” as a four-week advanced clerkship rotation. Inclusion health is an umbrella term describing people who experience social exclusion and who typically face multiple, overlapping risk factors for poor health, including poverty, violence, and complex psychosocial trauma. This paper is a case report of this innovative curriculum that empowers students enrolled in an undergraduate medical training program to address the health impacts of our evolving society at the population and individual levels.

## 2 Pedagogical frameworks

The curriculum of the Inclusion Health elective is grounded in five interrelated pedagogical frameworks that collectively support training future physicians to serve excluded populations competently. These include service-learning, problem-based learning (PBL), case-based learning (CBL), didactic instruction, and applied learning in cultural humility, and experiential learning in low-resource clinical environments. Together, they address the cognitive, affective, and situational dimensions of medical training to foster cultural humility, adaptability, self-efficacy, and the intent to serve marginalized communities. To provide greater specificity and support replicability, the learning objectives of the elective, along with their corresponding instructional methods and assessment strategies, are summarized in Table 1.

**Table 1:** Map of course objectives, teaching mode, and assessment.

Course objectives	Mode of teaching	Mode of assessment
Describe strategies to advocate for patients in collaboration with community partners	Lectures and discussion, case-based learning assignment	Assignment (disability determination letters)
Practice education of vulnerable patients and their families	Clinical interactions, lectures and discussion	Direct observation under clinical supervision, direct observation using standardized patient scenario
Develop and display cultural humility through compassionate communication in interactions with excluded patients and communities	Direct observation, lectures and discussion	Direct observation under clinical supervision, Direct observation using standardized patient scenario
Reflect on personal reactions in encountering vulnerable patients and emotionally charged situations	Clinical encounters, lectures and discussion	Assignment (reflective essay)
Demonstrate understanding of competing priorities and needs among excluded populations	Clinical encounters, lectures and discussion	Direct observation in classroom discussions, Direct observation under clinical supervision,
Identify risk and causal factors of health problems for patients in excluded populations	Direct observation, lectures and discussion	Assignment (reflective essay), direct observation under clinical supervision, Direct observation using standardized patient scenario
Identify and appraise services offered by agencies in Houston who serve excluded populations	Lectures, discussion, community projects	Direct observation in classroom discussions, direct observation under clinical supervision

Service-learning, deeply rooted in Dewey’s experiential learning philosophy, places students in community-based settings where clinical care is combined with critical reflection (10). This model ensures that service enriches learning and vice versa. Multiple studies affirm that service-learning enhances empathy, professional skills, and positive attitudes toward underserved groups, particularly those who experience poverty, homelessness, or social exclusion (11–13). In contexts similar to our elective, service-learning has been shown to reduce stigmas and stereotypes while embedding academic learning in socially meaningful engagement (12).

Problem-Based Learning integrates real-world problems into student-centered learning, cultivating clinical reasoning, self-directed inquiry, and teamwork. PBL improves problem-solving and communication skills and often yields equal or superior knowledge retention compared to traditional lecture-based methods (14). Furthermore, its constructivist orientation aligns with addressing complex SDOH; students must learn to integrate biomedical knowledge with psychosocial contextual understanding.

Case-Based Learning complements PBL by offering more structured clinical scenarios. Learners identify their own learning needs within real and realistic patient cases, bridging theoretical knowledge with clinical application. CBL enhances engagement, analytical ability, and motivation, all essential factors for responding to the intricate realities of marginalized patient care (15).

The didactic instruction and experiences focused on cultural humility offer a crucial scaffolding for every aspect of the elective. By intentionally teaching self-reflection, bias awareness, and inclusive communication, the course cultivates learners’ capacity for culturally informed care. Unlike competency-based educational models, humility emphasizes lifelong reflection and awareness of relational power (16). Structured curricula that promote awareness of societal inequities and systemic exclusion are essential for fostering sustained cultural reflectivity.

Experiential learning opportunities in low-resource, marginalized clinical settings bring the other frameworks into grounded practice. Immersion in environments such as clinics for the homeless, migrant health centers, and jail medicine facilitates the internalization of cultural humility, resourcefulness, and structural awareness. Role-modeling by clinicians experienced in community health, paired with reinforced didactic messaging and emotionally safe reflection, creates the conditions necessary for transformative learning (17, 18).

The design of the Inclusion Health elective intentionally interweaves these five frameworks. Service-learning situates students within community contexts, PBL and CBL deepen cognitive engagement through realistic scenarios, cultural humility instruction guides critical self-awareness, and the low-resource clinical immersion fosters adaptability and empathy. This integrated approach aims not only to build clinical and academic competencies but also to cultivate cultural humility, resourcefulness, and a long-term commitment to caring for excluded populations.

Together, these pedagogies reinforce each other. Service learning grounds moral engagement, while PBL and CBL sharpen problem-solving within, and even leveraging, socio-structural contexts. Dedicated cultural humility instruction provides reflective guardrails and increases self-awareness. Immersive, emotionally supportive clinical experiences anchor the other lessons in real-world practice. Such a comprehensive theoretical foundation positions the elective as a replicable, equity-oriented intervention in medical education, preparing future physicians to recognize, mitigate, and dismantle healthcare exclusion.

By integrating service learning with reflective practice, PBL, CBL, and focused didactics on cultural humility, all situated in real-world, resource-constrained care environments, this four-week elective aims to develop not only competence but also cultural humility and a sustained commitment to serving excluded populations. Each framework complements the others: service-learning grounds students in community realities; PBL and CBL build analytic and adaptive skills; dedicated cultural humility instruction fosters critical self-awareness; and the clinical setting ensures these lessons are emotionally resonant and contextually grounded.

## 3 Learning environment

The Inclusion Health elective at the TJFFCOM is a four-week rotation offered during the third phase of the medical school curriculum, focusing on tailored clinical exposure, structured didactics, and applied learning. The course immerses students in problem-based, applied learning activities and assignments centered on healthcare for populations experiencing homelessness and migration, equipping them with both hands-on experience and a theoretical foundation in SDOH.

Experiential learning forms the core of the elective, with students spending approximately 20 h per week in clinical rotations at sites that serve traditionally excluded patient populations. These settings might include Healthcare for the Homeless clinics, county jail primary care clinics, FQHCs, or charitable clinics that provide care to migrant and undocumented populations. Site preceptors, who hold voluntary faculty appointments with the College of Medicine, determine clinical schedules. To ensure a smooth transition into clinical environments, students receive a detailed four-week schedule in advance, including the specific time and location of their first clinical shift. This structure allows students to gain direct exposure to the complexities of healthcare delivery in resource-limited environments while engaging with patients facing significant barriers to care.

The didactic component complements clinical experiences through structured lecture days, held once per week, that incorporate active learning, discussions, and problem-based learning exercises. Topics covered in these sessions include behavioral health, substance use disorder treatment, integrated primary care, street medicine, and harm reduction. The integration of classroom-based learning with direct clinical exposure enables students to apply theoretical knowledge to real-world patient encounters and refine their approach to delivering equitable healthcare iteratively over the four-week rotation.

Students are also required to complete assignments designed to reinforce course content and enhance self-efficacy in addressing health disparities. These include a self-directed learning project, which allows students to independently explore a topic relevant to the elective, and a reflective essay in which they analyze their clinical experiences and discuss their impact on future medical practice. In addition to completing these assignments, students must meet specific course requirements, including attending all scheduled clinical shifts and lecture sessions, achieving a rating of “Meets Expectations” or higher on preceptor evaluations, and submitting a scholarly project based on course content.

The course follows a structured weekly format that balances patient care, independent study, and didactic learning. Each week, students dedicate half of their time to clinical rotations. The remainder of their schedule is divided between self-directed learning activities, including prework and course assignments, and a combination of asynchronous and synchronous lectures and discussions. To support clarity for readers and maintain flexibility across course iterations, the weekly structure, core themes, and expected time commitments are summarized in Table 2.

**Table 2:** Weekly schedule, student commitments, didactic focuses, clinical expectations, and assignments.

Week	Approximate time commitment	Core themes & didactic focus	Clinical expectations	Assignments/Assessments
Week 1	~20 hours clinical shifts 6–8 hours didactics (asynchronous + live) Independent study to reach ~40 hours total	Foundations of homelessness Social gradient of health Economics of homelessness and carceral systems Introductory case-based learning tied to initial clinical experiences	Introduction to clinical sites; care for individuals experiencing homelessness and migration	Begin reflective observations Participate in first CBL discussion
Week 2	~20 hours clinical shifts 6–8 hours didactics Independent study	Housing systems and social services Housing navigation, reentry, encampment engagement Role of community health workers	Continued on-site clinical immersion, focusing on navigating fragmented systems with patients	Applied assignment launched: disability determination assessment and physician-support letter (reviewed by course leadership)
Week 3	~20 hours clinical shifts 6–8 hours didactics Independent study	Behavioral health in excluded populations Mental health services Substance use treatment and harm reduction Overdose prevention Behavioral health integration	Clinical encounters emphasizing multimorbidity, behavioral health, and resource-limited care environments	Ongoing reflective synthesis Participation in behavioral-health-focused discussions
Week 4	~20 hours clinical shifts 6–8 hours didactics Independent study	Social drivers of health and medical complexity Respite care and non-medical drivers of health Preparing for ethical ambiguity in practice	Final clinical immersion; preparation for standardized patient encounter	Standardized patient assessment in simulation lab Final reflective essay and debrief session

The first week of the elective introduces students to foundational concepts such as homelessness, the social gradient of health, and the economics of homelessness, carceral systems, alongside a case-based learning exercise drawn from their initial clinical experiences.

The second week shifts focus to housing systems and social services, covering topics such as housing navigation, reentry, the role of community health workers, encampment engagement strategies, and launches the first applied learning assignment where students complete a disability determination assessment and develop a physician letter (reviewed and finalized by course leadership) supporting an application for disability supplemental income.

In the third week, students explore behavioral health considerations, including mental health services, substance use treatment, overdose prevention, and the integration of behavioral health into primary care.

The final week addresses social drivers of health and medical complexity, with discussions on multimorbidity, respite care, and non-medical drivers of health, culminating in a standardized patient assessment and a concluding reflection session. The patient encounter is held in a simulation lab and observed by a family medicine physician (co-course director) with extensive experience in delivering healthcare to excluded populations. The standardized patient assessment is designed to challenge students with an unresolvable or potentially uncomfortable clinical situation (differentiating it from prior standardized, simulated encounters) and evaluate the students’ cultural humility, communication skills, and empathy.

## 4 Assessment

Since its inception in 2023, the Inclusion Health elective has been completed twice, with a 100% assignment completion rate. Clinical preceptor evaluations have been uniformly positive, with all students receiving ratings of “Exceeds Expectations.” Qualitative feedback from students has reinforced the impact of the course, described once as the most meaningful experience of their medical education. Several students have expressed interest in repeating the elective in a later block, further underscoring its value.

A novel and thorough rubric was developed for the standardized patient assessment to support the final formative assessment. This rubric provides space for structured evaluation and rich, qualitative feedback from the observing attending physician. The emphasis on narrative suggestions ensures that student learning is not only graded but meaningfully contextualized. In addition to faculty assessments, a form for individualized, qualitative feedback was created for patients to communicate perceptions of the student encounter. This inclusion of patient voice in the evaluation process aligns with the course’s broader goal of fostering cultural humility and empathetic care.

The initial standardized patient assessment has been identified as an area for refinement and expansion. While currently used solely for individualized feedback provided by the course co-director, broader validation and adaptation could enhance its applicability and ensure replicability in other academic settings. Ongoing efforts are focused on refining this assessment to improve its utility for formative and summative evaluation, further strengthening the course’s role in preparing future physicians to address healthcare disparities among excluded populations. Simultaneously, the development of additional complex and realistic standardized patient cases is underway, which will support the expansion of this evaluation method beyond the elective and potentially into earlier stages of medical training for all students, not just elective participants.

Looking forward, the course will continue to track key outcomes for student participants. These include academic and professional milestones such as graduation rates, residency match outcomes, and specialty selection. Plans are in development for an annual post-graduation survey to assess participants’ involvement in occupational or volunteer work with inclusion health populations. These longitudinal data will help determine whether the course fulfills its goal of preparing physicians who are not only clinically competent but also equipped with the mindset and tools necessary to provide principled care to patients who are routinely excluded from traditional primary healthcare systems.

## 5 Discussion on the practical implications, objectives, and lessons learned

This structured approach: integrating service learning, problem-based and case-based learning, didactic instruction in cultural humility, and immersive experiences in low-resource clinical environments, fosters professional development, clinical competency, and a deeper understanding of systemic inequities affecting vulnerable patient populations. The Inclusion Health elective exposes learners to the medical complexity and SDOH faced by excluded populations. It provides the reflective and experiential tools necessary to address these challenges with empathy and adaptability.

People experiencing homelessness have much to teach us about cultural humility. Many have endured profound deprivation, disproportionate exposure to trauma, and repeated encounters with structural violence as a consequence of having little or no power in society (19, 20). Cultural humility in healthcare demands more than knowledge of diversity; it requires a lifelong commitment to self-evaluation, self-critique, and the active redressing of power imbalances (6). Through intentional practice, physicians can shift from a “fixer” mindset to a collaborative stance that honors patients as experts in their own lives (5).

The challenge in medical education is to balance the confidence needed for competent practice with humility and reflective capacity. This elective’s layered design—combining authentic community engagement with structured reflection, skill development in behavioral health, and standardized patient encounters—helps learners build a portfolio of experiences that promote a sustainable, reflective professional identity.

By confronting learners with unresolvable or ethically ambiguous scenarios, the standardized patient assessments provide a psychologically safe space to practice navigating discomfort, exploring shared decision-making, and acknowledging uncertainty. These experiences reinforce the skills necessary for effective partnerships in care, especially in settings where structural and social barriers complicate the clinical encounter.

Ultimately, understanding power dynamics in patient–physician relationships is essential for advancing health equity. Opportunities for role flexibility, power-sharing, and authentic partnership can foster trust and encourage behavior change: critical elements in improving health outcomes among populations who have been historically excluded from traditional primary care systems (21, 22). Longitudinal follow-up of elective participants will determine whether such educational strategies translate into sustained professional engagement with inclusion health populations, offering a model for replication in other medical education settings.

## 6 Acknowledgment of constraints

As with many educational interventions embedded within the rigid structure of medical training, the Inclusion Health elective is subject to several limitations that warrant consideration. Chief among these is the time-limited nature of the course. Structured as a four-week elective during the third phase of the medical school curriculum, the elective operates within a narrow window of opportunity to engage students in the breadth and complexity of inclusion health. While this duration allows for concentrated exposure to systematically excluded populations and targeted didactics, it inevitably constrains the depth and continuity of clinical immersion and skill development. Clinical engagement is maintained at approximately 24 h per week to meet elective credit requirements while preserving time for didactic instruction and self-directed learning, but this compromises prolonged or longitudinal patient interactions that are often central to cultivating cultural humility and sustained insight.

Moreover, the elective competes with a range of other clinical and research opportunities in the final phase of medical school—many of which are perceived as more directly aligned with securing residency placements. As a result, self-selection bias may influence enrollment, with students who already possess a strong interest in health equity or social justice being more likely to participate. This selection bias poses limitations on generalizability and may reduce the opportunity to reach students who might benefit most from this content.

From an operational standpoint, resource limitations are also a critical factor. The course is facilitated mainly through voluntary contributions of time and expertise by community-based preceptors and faculty with specialized experience in caring for excluded populations. While this approach strengthens community partnerships and reinforces the elective’s mission, it also renders the course vulnerable to fluctuations in capacity, availability, and institutional support. These limitations—time, competition, resource constraints, and selection effects—underscore the challenges of implementing transformative educational experiences in constrained academic environments.

## 7 Conclusion

Ultimately, the most critical outcome is the cultivation of cultural humility among participants. By presenting students with a structured, digestible, and clinically integrated experience of healthcare for marginalized populations, the elective aims to instill lasting attitudes and approaches that prioritize equity, inclusion, and patient-centered care. As participants in the elective graduate from their undergraduate medical education and move on to internships and residencies, the hope is that they are more compassionate toward complex patients, more able to address psychosocial and structural barriers to health, and more inclined and capable of working in nontraditional care environments.

## Data Availability

The original contributions presented in the study are included in the article/supplementary material, further inquiries can be directed to the corresponding author.
